# The CaCA superfamily genes in *Saccharum*: comparative analysis and their functional implications in response to biotic and abiotic stress

**DOI:** 10.1186/s12864-021-07828-3

**Published:** 2021-07-18

**Authors:** Weihua Su, Chang Zhang, Dongjiao Wang, Yongjuan Ren, Tingting Sun, Jingfang Feng, Yachun Su, Liping Xu, Mutian Shi, Youxiong Que

**Affiliations:** 1 Key Laboratory of Sugarcane Biology and Genetic Breeding, Ministry of Agriculture, Fujian Agriculture and Forestry University, 350002 Fuzhou, Fujian China; 2 Key Laboratory of Genetics, Breeding and Multiple Utilization of Crops, Ministry of Education, College of Crop Science, Fujian Agriculture and Forestry University, 350002 Fuzhou, Fujian China; 3 College of Horticulture, Fujian Agriculture and Forestry University, 350002 Fuzhou, Fujian Province China

**Keywords:** Ca^2+^/cation antiporter (CaCA) superfamily, *Saccharum*, Molecular evolution, Functional divergence, Stress, Subcellular location

## Abstract

**Background:**

In plants, Calcium (Ca^2+^) acts as a universal messenger in various signal transduction pathways, including responses to biotic and abiotic stresses and regulation of cellular and developmental processes. The Ca^2+^/cation antiporter (CaCA) superfamily proteins play vital roles in the transport of Ca^2+^ and/or other cations. However, the characteristics of these superfamily members in *Saccharum* and their evolutionary and functional implications have remained unclear.

**Results:**

A total of 34 *CaCA* genes in *Saccharum spontaneum*, 5 *CaCA* genes in *Saccharum* spp. R570, and 14 *CaCA* genes in *Sorghum bicolor* were identified and characterized. These genes consisted of the H^+^/cation exchanger (CAX), cation/Ca^2+^ exchanger (CCX), EF-hand / CAX (EFCAX), and Mg^2+^/H^+^ exchanger (MHX) families, among which the CCX and EFCAX could be classified into three groups while the CAX could be divided into two groups. The exon/intron structures and motif compositions suggested that the members in the same group were highly conserved. Synteny analysis of *CaCAs* established their orthologous and paralogous relationships among the superfamily in *S. spontaneum*, R570, and *S. bicolor*. The results of protein-protein interactions indicated that these CaCA proteins had direct or indirect interactions. Quantitative reverse transcription polymerase chain reaction (qRT-PCR) analysis demonstrated that most members of *Saccharum CaCA* genes exhibited a similar expression pattern in response to hormonal (abscisic acid, ABA) treatment but played various roles in response to biotic (*Sporisorium scitamineum*) and abiotic (cold) stresses. Furthermore, *ScCAX4*, a gene encoding a cytoplasm, plasma membrane and nucleus positioning protein, was isolated from sugarcane. This gene was constitutively expressed in different sugarcane tissues and its expression was only induced at 3 and 6 h time points after ABA treatment, however was inhibited and indued in the whole process under cold and *S. scitamineum* stresses, respectively.

**Conclusions:**

This study systematically conducted comparative analyses of *CaCA* superfamily genes among *S. spontaneum*, R570, and *S. bicolor*, delineating their sequence and structure characteristics, classification, evolutionary history, and putative functions. These results not only provided rich gene resources for exploring the molecular mechanism of the *CaCA* superfamily genes but also offered guidance and reference for research on other gene families in *Saccharum*.

**Supplementary Information:**

The online version contains supplementary material available at 10.1186/s12864-021-07828-3.

## Background

Calcium (Ca^2+^) is a universal ion that exists in all organisms as a critical element and an essential nutrient and also functions as a ubiquitous secondary messenger [1, 2]. There are several particularly important transporters that act as “gatekeepers”, mediating the movement of Ca^2+^. Previous studies showed that three classes of membrane transporters, Ca^2+^-ATPases (PMCAs), Ca^2+^ permeable channels, and Ca^2+^/cation antiporters (CaCAs), act as “gatekeepers” to mediate Ca^2+^ flux across the membrane and to regulate cytosolic Ca^2+^ levels [3–5].

CaCA superfamily proteins are widespread in archaea, bacteria, fungi, plants and animals [6, 7]. They can enhance the efflux of Ca^2+^ across membranes against the concentration gradient by exchanging the influx of monovalent cations such as H^+^, Na^+^, or K^+^ to energize the process [6–8]. As a superfamily, CaCAs consist of a number of exchanger protein families [7]. According to a study by Cai et al. [7], the CaCA superfamily can be classified into six families, i.e., the YRBG, Na^+^/Ca^2+^ exchanger (NCX), Na^+^/Ca^2+^, K^+^ exchanger (NCKX), cation/Ca^2+^ exchanger (CCX), and H^+^/cation exchanger (CAX) families.

As previous studies have shown, YRBG family proteins are present in many prokaryotes but are absent in eukaryotes [7, 9]. Regarding the NCX and NCKX families, they are primarily present in animal groups [7]. Due to the speed and high capacity for Ca^2+^ in the NCX family, NCXs are important regulators of cellular Ca^2+^ homeostasis [8]. In mammals, the NCX exchange proteins consist of three distinct types (NCX1, NCX2 and NCX3) [8]. Plants have evolved a novel CaCA group, the Mg^2+^/H^+^ exchanger (MHX) proteins, which belong to the NCX family [8, 10, 11]. The CAX protein family has been observed in various organisms including bacteria, protozoa, fungi, animals, algae, and plants [8, 12–14]. Normally, the CAX family is divided into three types: 1, 2, and 3 [12]. In addition, a novel group of EF-hand / CAX (EFCAX) proteins containing EF-hand domain which are also termed as NCX-like proteins (NCL), has been identified in the CAX family [8]. This novel group is evolutionarily closer to CAX proteins than NCX proteins [8, 15]. Furthermore, functional characterization demonstrated that AtNCL exhibited Na^+^/Ca^2+^ exchange activity [16].

*Saccharum* spp. (sugarcane), an important sugar and biofuel feedstock crop, accounts for 80 % of the world’s total sugar production and provides 40 % of bio-ethanol [17, 18]. At present, various stresses, are the main factors that restrict the well development of sugar industry [19]. For example, it is manifested that salt stress cause considerable reduction in growth rate at various sugarcane growth stages [20]. Under cold and drought stresses, the photosynthetic rate of sugarcane is severely reduced [19, 21]. In order to avoid the negative effects of stresses, plants have evolved complex mechanisms, such as osmotic adjustment [22] which is mainly dependent on the regulation of inorganic ions (Na^+^, K^+^, Ca^2^^+^, and Cl^−^) [23]. Previous studies have demonstrated that CaCAs are essential for controlling ion concentrations to maintain cellular functions [13, 24]. However, no comprehensive and systematic research on the CaCA superfamily was previously conducted in *Saccharum*. Herein, two currently available *Saccharum* species genomes, R570 (*Saccharum* spp., the haploid genome of the modern sugarcane cultivar) [25] and AP85-441(*Saccharum spontaneum*, the sugarcane ancestor) [17] as well as the representative genome of the closest relative (*Sorghum bicolor*) [26] were selected to perform genome-wide identification and comprehensive characterization of CaCA proteins in *Saccharum*. The phylogenetic relationships, gene and protein characteristics, duplication events, and synteny relationships were further used to investigate the evolutionary relationships of *CaCA* genes. The interactive relationships between *CaCAs* and microRNAs, gene ontology annotation, and protein interactions of CaCA proteins and their expression patterns in response to hormonal (abscisic acid, ABA), biotic (*Sporisorium scitamineum*), and abiotic (cold) stresses were also evaluated. Furthermore, one *CAX* gene was isolated from sugarcane, and its expression patterns and subcellular localization were analyzed. The present study is expected to support a theoretical basis for further investigations of the clear functions of *CaCA* genes in *Saccharum*.

## Results

### Identification and sequence features of ***CaCA*** genes in ***S. spontaneum***, R570 and ***S. bicolor*** genomes

**Fig. 1:**
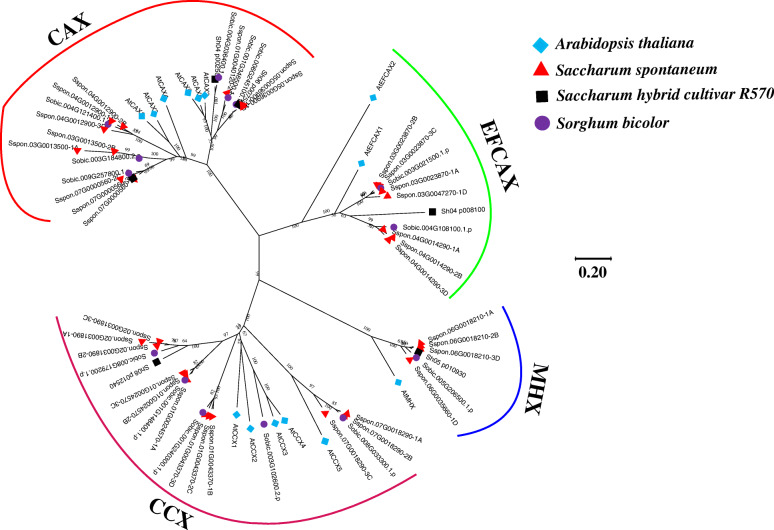
Phylogenetic analysis of the *CaCA* genes from *A. thaliana*, *S. spontaneum*, R570, and *S. bicolor*

Statistical results showed that 34 copies of *CaCA* genes were present in *S. spontaneum*, with 14 copies in *S. bicolor*, while R570 had only five *CaCA* genes. To reveal the taxonomic information of *CaCA* superfamily genes, a phylogenetic tree based on the amino acid homology among *Arabidopsis*, *S. spontaneum*, R570, and *S. bicolor* was constructed using the neighbor-joining (NJ) method (Fig. 1). The phylogenetic tree indicated that *S. spontaneum* possessed 11 *CAX* genes, 12 *CCX* genes, 7 *EFCAX* genes, and four MHX genes. In R570, two copies of *CAX* genes and only one *CCX* gene, one *EFCAX* gene, and one *MHX* gene were identified. In *S. bicolor*, there were six *CAX* genes, five *CCX* genes, two *EFCAX* genes, and one *MHX* gene.

The physical and chemical parameters of these CaCA proteins were computed using the ExPASy ProtParam tool (Supplemental Figure S1, Supplemental Table S1 and Table S2). Comparative analysis showed that the number of amino acid residues spanned the largest range in SsCaCA proteins, from 247 in SsCCX4c to 1214 in SsEFCAX2. The number of amino acid residues ranged from 347 (ShEFCAX1) to 641 (SbCCX3) in ShCaCAs and SbCaCAs, respectively. The computed theoretical isoelectric points indicated that the acidity or alkalinity of CaCAs varied greatly in *Saccharum* and *S. bicolor*. The results also suggested that these CaCAs in *S. spontaneum*, R570, and *S. bicolor* contained at least five transmembrane domains, most of which were located in the plasma membrane.

### Phylogenetic classification of the CaCA superfamily

**Fig. 2:**
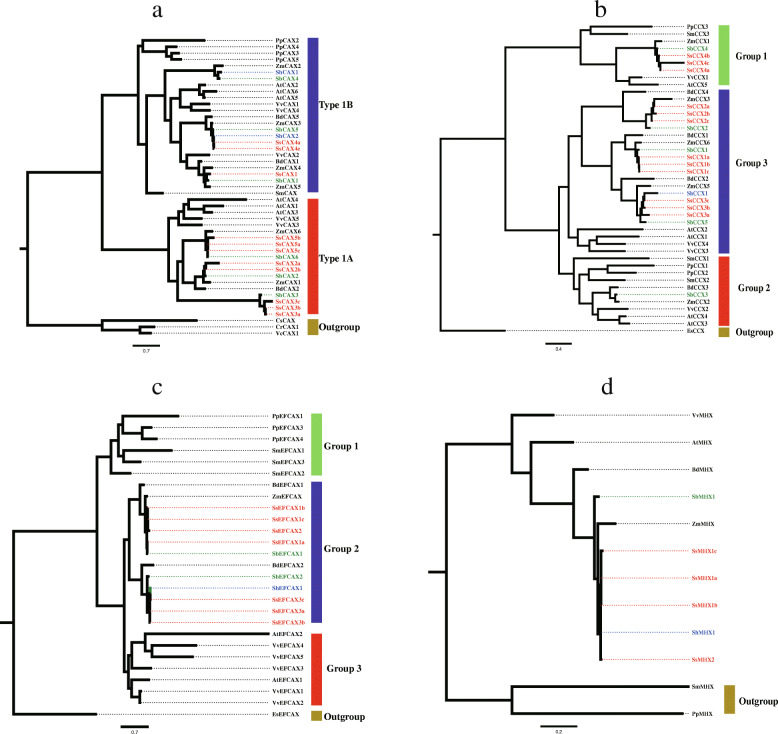
Phylogenetic evolutionary tree of the CaCA superfamily members. (**a**) An NJ phylogenetic tree was constructed using the full-length sequence alignments of 47 CAX proteins identified using MUSCLE in MEGAX. (**b**) An NJ phylogenetic tree was constructed using the full-length sequence alignments of 43 CCX proteins identified using MUSCLE in MEGAX. (**c**) An NJ phylogenetic tree was constructed using the full-length sequence alignments of 28 EFCAX proteins identified using MUSCLE in MEGAX. (**d**) An NJ phylogenetic tree was constructed using the full-length sequence alignments of 12 MHX proteins identified using MUSCLE in MEGAX. All SsCaCA, ShCaCA, and SbCaCA proteins are highlighted in red, blue, and green, respectively. All the corresponding reference numbers are listed in Supplemental Table S1 and Table S3

The phylogenetic tree, which was based on comparing the amino acid sequences among algae, mosses, monocots, and dicots, was constructed using the NJ and maximum likelihood (ML) methods to unveil the CaCA superfamily functional information (Fig. 2 and Supplemental Figure S2). In generally, the topologies of the NJ and ML trees constructed in this study were highly consistent, demonstrating the reliability of our classification. In the CAX family, 19 CAX (11 SsCAXs, two ShCAXs, and six SbCAXs) proteins could be divided into two groups (Type 1A and Type 1B). The Type 1B group contained CAX members from mosses, monocots, and dicots, while the Type 1A group only contained CAX members from monocots and dicots. Within the Type 1A group, there was a clear distinction between the genes from monocot and dicot plants, though this division was not as obvious as that within the Type 1B group. In the CCX family, 18 CCXs (12 SsCCXs, one ShCCX, and five SbCCXs) could be classified into three groups (Group 1, Group 2, and Group 3). A clear distinction between the proteins from monocot and dicot plants was also observed among these three groups. Interestingly, the EFCAX family was clearly clustered into three major groups (Group 1, Group 2, and Group 3), which corresponded to mosses, monocots, and dicots, respectively. Ten EFCAXs (seven SsEFCAXs, one ShEFCAX, and two SbEFCAXs) were all sorted into the monocot group, which was also named Group 2. In the MHX family, except for the two MHX members from mosses, the other MHXs from monocots and dicots were on the same branch. It should be noted that six MHXs, i.e., four SsMHXs, one ShMHX, and one SbMHX, had closer relationships with ZmMHX.

### Protein motifs and gene structure analysis

A total of 10 distinct conserved motifs found in each species are illustrated in Supplemental Figure S3. Whether in the CAX, CCX, EFCAX, or MHX family, most members belonged to the same group and shared common motif compositions. What should also be stressed here is that, even in the same classification, the motifs of some proteins were unique. For example, compared with the other CAXs, SsCAX3c contained double motifs 1, 2, 3, 4, 5, 7, and 9. *ScCAX4e* was the duplicated gene of *ScCAX4a*, and motif 4 was lost in ScCAX4e. Compared with SbCCX4, SsCCX4a, SsCCX4b, and SsCCX4c, the motifs 2, 4, 5, 6, and 10 were lost in SsCCX4c and motif 6 was lost in ScCAX4a. In the EFCAX family, SsEFCAX2 had the largest number of motifs, containing double motifs 2, 3, 4, 5, 6, 7, 8, 9, and 10, while ShEFCAX1 only had six motifs. It is interesting that all of the MHX proteins contained the same motif composition, expect for SsMHX2.

As exhibited in the pattern of exon–intron distribution and the position of all *CaCA* genes, the genes from the *CCX* family were intron-poor with < 3 introns. It was notable that those closely related genes were usually more similar in gene structure. For instance, *SsEFCAX1a*, *SsEFCAX1b*, and *SsEFCAX1c* all had six introns. However, some closely related genes showed significant differences in structural arrangements. For example, *SsCAX3a* possessed 11 introns and *SsCAX3b* had eight introns, while *SsCAX3c*, a closely related gene, had 19 introns. Intriguingly, all *MHX* genes contained seven introns in the three studied species (*S. spontaneum*, R570 and *S. bicolor*).

### Chromosomal distribution, duplications, and synteny analysis of the CaCA superfamily

**Fig. 3:**
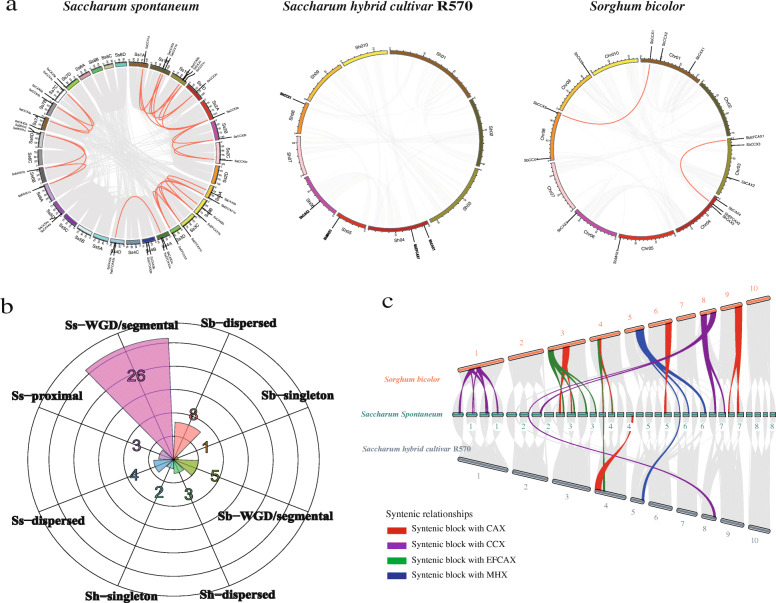
Duplication events of *CaCA* genes in *S. spontaneum*, R570, and *S. bicolor*. (**a**) Mapping of *CaCA* genes and the duplications among them on the *S. spontaneum*, R570, and *S. bicolor* chromosomes. Gray lines indicate all syntenic blocks in the *S. spontaneum*, R570, and *S. bicolor* genome. The red lines indicate collinear relationships among *CaCA* genes. The chromosome number is indicated at the top of each chromosome (**b**) Distribution of gene type among *CaCA* genes in *S. spontaneum*, R570, and *S. bicolor*. (**c**) Syntenic relationships of *S. spontaneum*, R570, and *S. bicolor* genes among *S. spontaneum*, R570, and *S. bicolor*

The chromosomal distribution showed that 34 *SsCaCA*, five *ShCaCA*, and 14 *SbCaCA* genes were unevenly distributed on 20, 4, and 7 numbers of chromosomes, respectively. Expect for *ShCaCAs*, there were 25 and two duplicated *SsCaCA* gene pairs in the *S. spontaneum* and *S. bicolor* genomes, respectively (Fig. 3a, Supplemental Table S5).

To elucidate the evolutionary genome rearrangement and duplication patterns of the CaCA protein encoding genes in *S. spontaneum*, R570, and *S. bicolor*, an analysis of gene duplication events including whole genome duplications (WGD)/segmental, dispersed duplication, proximal duplication, singleton duplication, and tandem duplication was performed (Fig. 3b, Supplemental Table S6). Duplication was observed in all predicted *CaCA* genes, among which WGD/segmental duplications were the main modes in *SsCaCAs*, while dispersed duplications were the main modes in *ShCaCAs* and *SbCaCAs* (Fig. 3b).

In order to further infer the evolutionary mechanism of *CaCA* superfamily genes, syntenic maps between *S. bicolor*, R570, and *S. spontaneum* were constructed (Fig. 3c). As shown in Fig. 3c, only four orthologous pairs between *S. spontaneum* and R570 were found. Between *S. spontaneum* and *S. bicolor*, 27 syntenic orthologous gene pairs were observed. We found that one *S. bicolor* gene corresponded to multiple *S. spontaneum* genes, such as *SbCCX1* - *SsCCX1a*/*1b*/*1c*. A comparison of the syntenic blocks showed that 19 collinear gene pairs, 18 pairs between *S. bicolor* and *S. spontaneum* and one pair between *S. bicolor* and R570, were anchored to the highly conserved syntenic blocks, which spanned more than 100 genes. Only three collinear gene pairs (*SbCAX3*-*SsCAX3b*, *SbCCX1*-*SsCCX1b*, and *SbCCX5*-*SsCCX3b*) were located in syntenic blocks that possessed fewer than 30 orthologous gene pairs (Supplemental Table S7).

According to the syntenic relationships of *CaCA* genes from *S. spontaneum*, R570, and *S. bicolor*, the synonymous (Ks), non-synonymous (Ka), and Ka/Ks ratio values were calculated (Supplemental Table S7). The Ka/Ks ratio showed that all Ka/Ks values of the orthologous *CaCA* genes among *S. spontaneum*, R570, and *S. bicolor* were < 1, suggesting that these orthologous genes underwent strong purifying selection for retention.

### microRNA target prediction

In order to reveal the interactions between microRNAs (miRNAs) and their *CaCA* gene targets, the potential networks were predicted by the psRNATarget server (Supplemental Figure S4 and Supplemental Table S8). In *S. spontaneum*, four *SsCAXs* and three *SsCCXs* were regulated by four miRNAs. It is worth noting that *ShCAX1* has nine miRNA target sites in two miRNA families. Surprisingly, seven *SbCaCA* genes, i.e., two *SbCAXs*, four *SbCCXs*, and one *SbMHX*, were regulated by 49 miRNAs. In general, one *CaCA* gene might be targeted by multiple miRNAs, while several *CaCA* genes might be regulated by the same miRNA.

### Gene ontology (GO) annotation

GO annotation was performed for all *CaCA* genes to determine their potential functions. As shown in Supplemental Figure S5, *CaCA* genes are involved in various biological processes (BP), molecular functions (MF), and cellular components (CC) (Supplemental Table S9). Under the BP category, we found that all of the *CaCA* genes (53) were further annotated to localization and cellular processes, while 28 were annotated to biological regulation, 10 to response to stimulus, and two to metabolic processes. In the MF category, they were annotated to transporter activity (33 genes), binding (10 genes), and catalytic activity (two genes), which agreed well with the transporter property of these *CaCA* genes. With respect to the CC category, the majority of *CaCA* genes were predicted to be involved in the cellular anatomical entity (39 genes) and intracellular (38 genes) categories. In addition, 28 *CaCA* genes were involved in the cell category and two *CaCA* genes encoded protein-containing complexes.

### Interactions among CaCA proteins

**Fig. 4:**
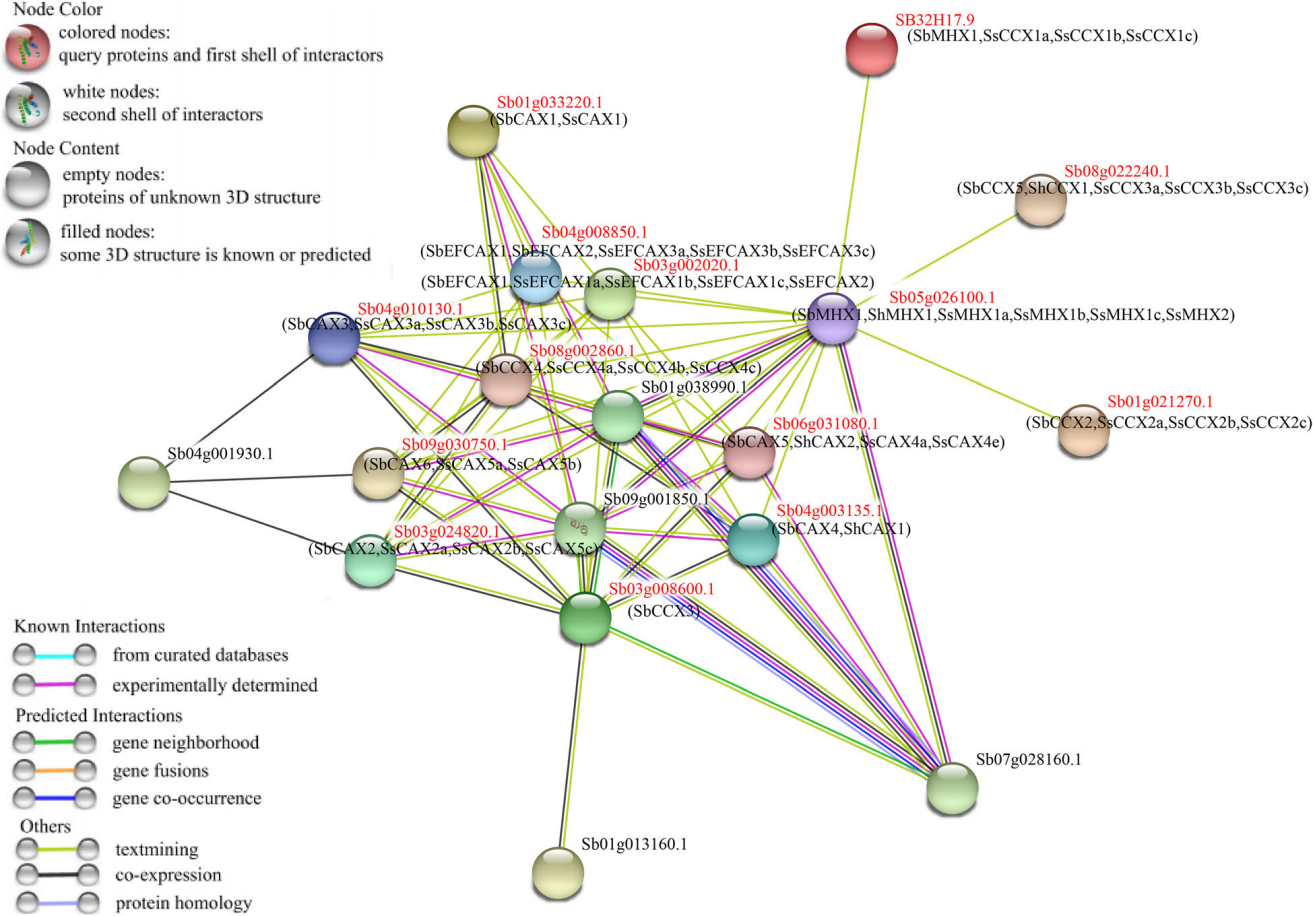
Predicted protein–protein interactions of CaCAs according to their orthologs in *S. bicolor*. In the network, only the pairs with more than 60 % sequence identity between SbCaCAs, ShCaCAs, or SsCaCAs and SbCaCAs and with an interaction score > 0.4 are shown. Line and node colors indicate the different types and degrees of interactions, respectively. The filled or empty nodes represent known or unknown 3D structures, respectively. The gene names in parentheses indicate that paralogous or orthologous gene names

Predicting the interactions among CaCA proteins is helpful for understanding their interactive relationships. As shown in Fig. 4, a total of 53 CaCA proteins were predicted to have direct or indirect interaction relationships. For example, Sb09g030750.1 was predicted to have direct interactions with Sb05g026100.1, Sb03g008600.1, Sb04g008850.1, Sb01g033220.1, or Sb08g002860.1. It is worth noting that these CaCA proteins may interact with the peroxisome biogenesis protein (Sb09g001850.1), plasma-membrane choline transporter (Sb01g013160.1), plasma membrane-type calcium-transporting ATPase 2 (Sb07g028160.1), and endoplasmic reticulum-type calcium-transporting ATPase 4 (Sb01g038990.1 and Sb09g001850.1). In general, these interactive relationships provide an important reference for identifying the true interactions of CaCA proteins in biochemical experiments.

### Expression profiles of ***CaCA*** genes in sugarcane in response to hormonal (ABA) stress

**Fig. 5:**
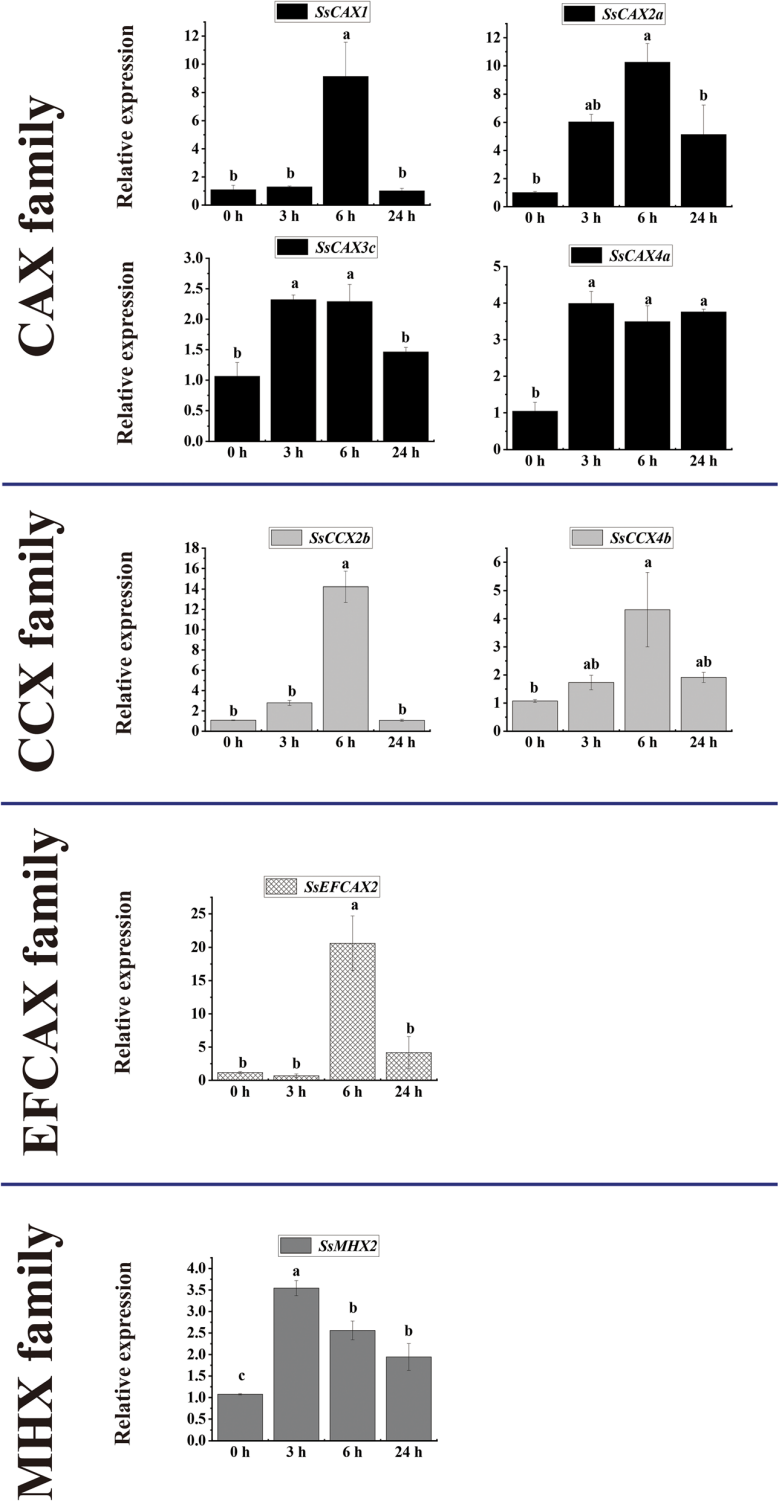
Expression dynamics of the candidate *CaCA* genes in sugarcane in response to ABA treatment. Error bars represent the standard error (SE) of three independent biological experimental repeats. The value on the Y-axis indicates the relative gene expression levels. The x-axis represents the time points when the samples were collected. Different lowercase letters indicate a significant difference, determined by one-way ANOVA, followed by Duncan’s new multiple range test (*p* < 0.05)

Eight *CaCA* genes were retained for the quantitative reverse transcription polymerase chain reaction (qRT-PCR) analysis. The expression profiles of eight *CaCA* genes in sugarcane under ABA treatment were successfully detected (Fig. 5). In brief, all *CaCA* genes were induced at 6-h time points, and five *CaCA* genes from the *CAX*, *CCX*, and *EFCAX* families peaked at 6 h post-treatment. Five *CaCA* genes (*SsCAX2a*, *SsCAX3c*, *SsCAX4a*, *SsCCX4b*, and *SsMHX2*) were induced at both 3 h and 6 h.The transcript profiles of *SsCAX2a*, *SsCAX4a*, *SsCCX4b*, and *SsMHX2* were promoted at all treated time points.

### Expression characteristics of ***CaCA*** genes in sugarcane under biotic (***Sporisorium scitamineum***) stress

**Fig. 6:**
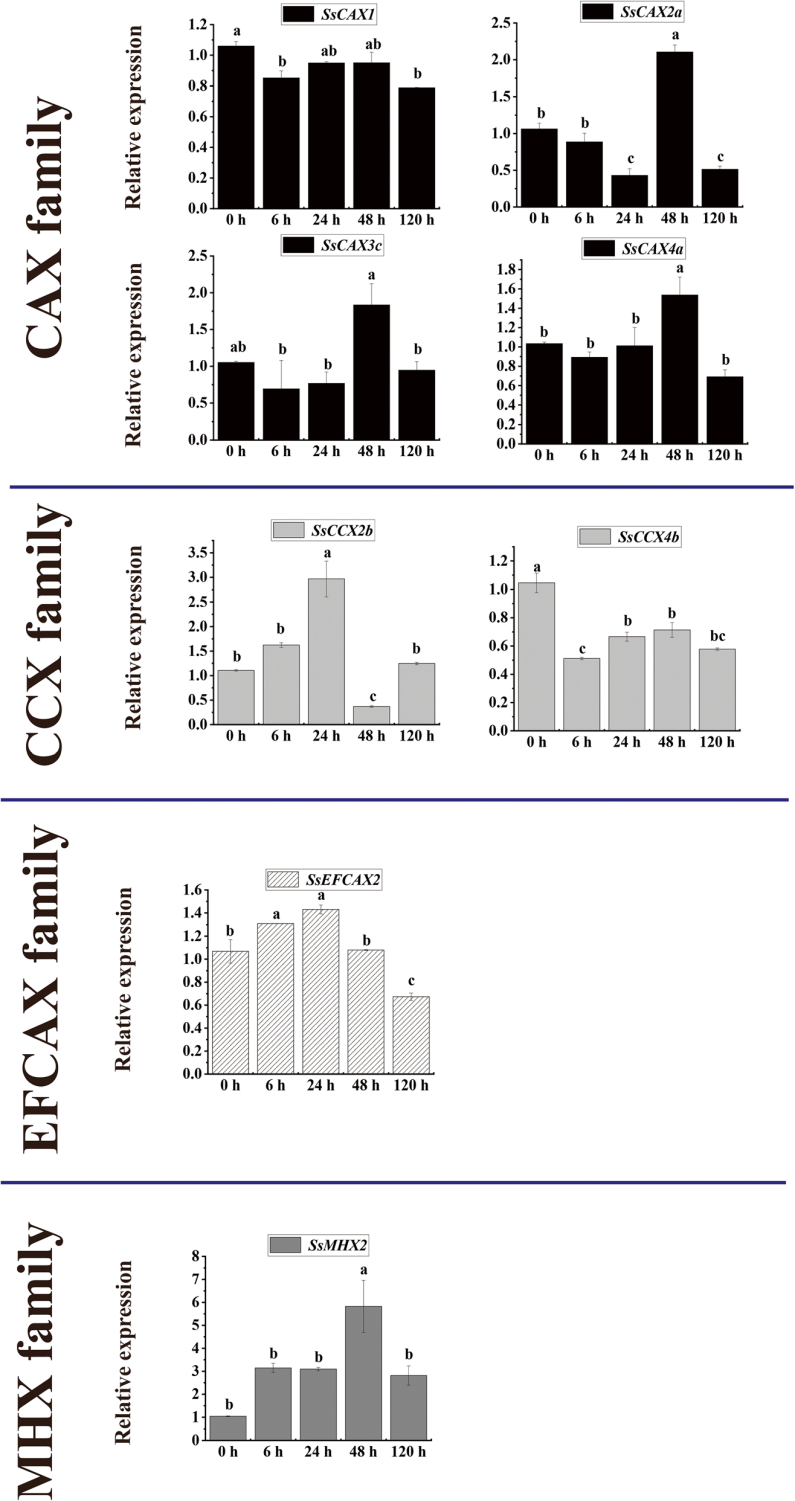
Expression dynamics of the candidate *CaCA* genes in sugarcane after *Sporisorium scitamineum* infection. Error bars represent the standard error (SE) of three independent biological experimental repeats. The value on the Y-axis indicates the relative gene expression levels. The x-axis represents the time points when the samples were collected. Different lowercase letters indicate a significant difference, determined by one-way ANOVA, followed by Duncan’s new multiple range test (*p* < 0.05)

qRT-PCR analysis was performed to investigate the expression characteristics of eight *CaCA* genes in sugarcane in response to *S. scitamineum* (Fig. 6). In the *CAX* family, the expression of *SsCAX1* was inhibited at all treatment time points. Three *CAX* genes (*SsCAX2a*, *SsCAX3c,* and *SsCAX4a*) had the highest expression at 48 h. In the *CCX* family, *SsCCX4b* were downregulated at all treatment time points. At 24 h, *SsCCX2b* had the highest expression levels. The expression of *SsEFCAX2* was upregulated at 6 and 24 h, and downregulated at 120 h. The expression level of *SsMHX2* was upregulated at 48 h.

### The abiotic (cold) stress-induced expression profiles of ***CaCA*** genes in sugarcane

**Fig. 7:**
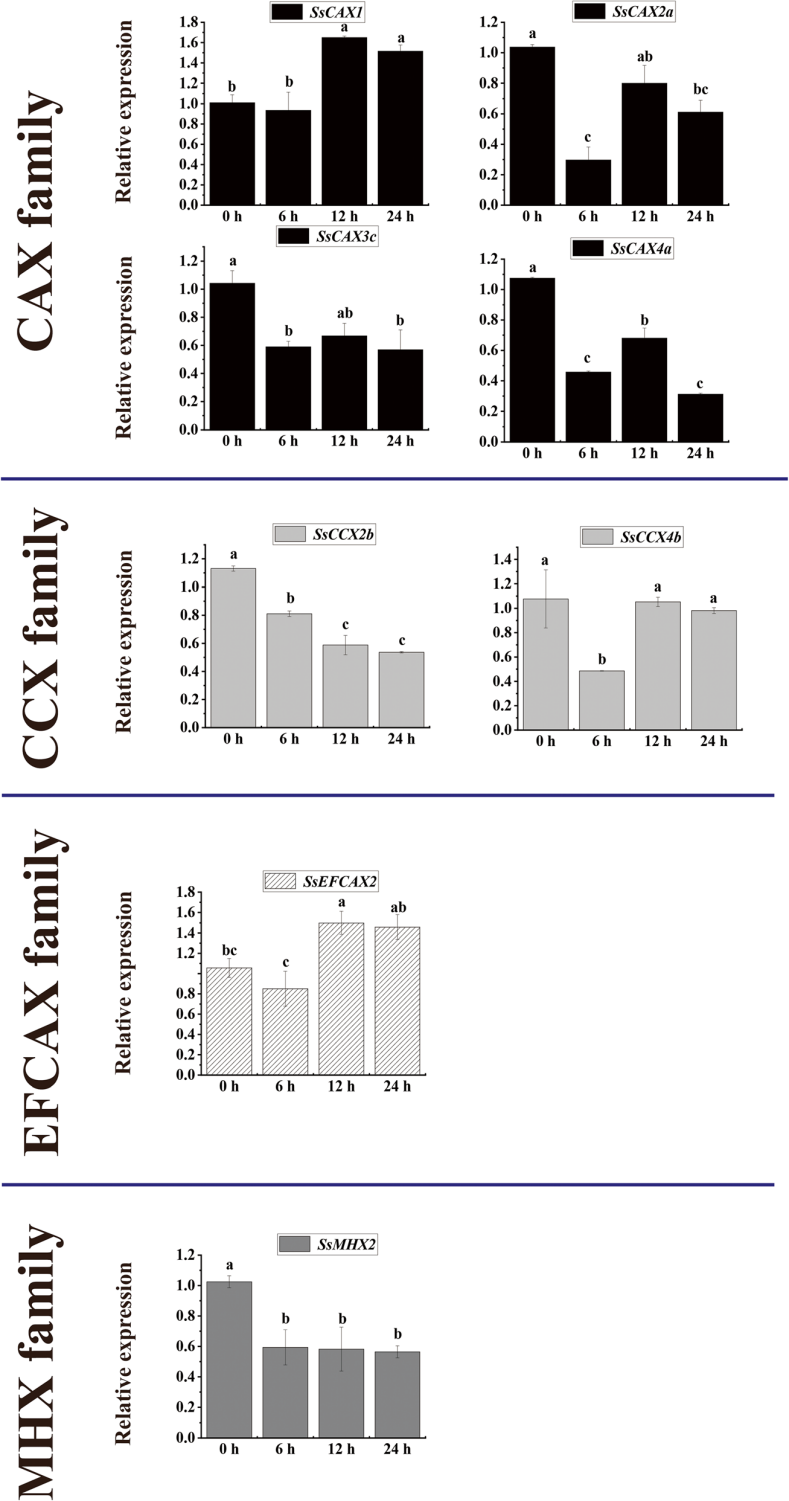
Expression dynamics of the candidate *CaCA* genes in sugarcane under cold stress. Error bars represent the standard error (SE) of three independent biological experimental repeats. The value on the Y-axis indicates the relative gene expression levels. The x-axis represents the time points when the samples were collected. Different lowercase letters indicate a significant difference, determined by one-way ANOVA, followed by Duncan’s new multiple range test (*p* < 0.05)

The transcriptional profiles of eight *CaCA* genes under cold stress were monitored by qRT-PCR in this study (Fig. 7). In the *CAX* family, the expression of *SsCAX1* was upregulated at 12 and 24 h. Under cold stress, three *CAX* genes were downregulated at all treatment time points. In the *CCX* family, *SsCCX2b* were downregulated at all treatment time points and the expression levels of *SsCCX4b* were inhibited at 6 h. *SsEFCAX2* was upregulated at 12 and 24 h. The expression levels of *SsMHX2* were downregulated at all treatment time points.

### Identification and sequence analysis of ***ScCAX4***

**Fig. 8:**
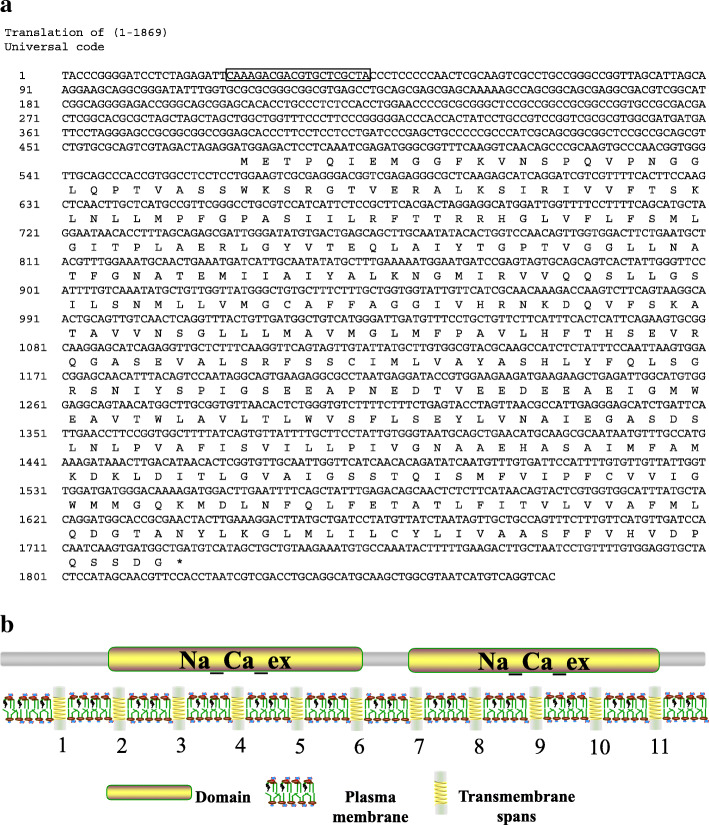
The sequence of the *ScCAX4* gene and the characteristics of its encoded protein. (**a**) Complete cDNA and deduced amino acid sequences of the *ScCAX4* gene. The full length cDNA was 1251 bp with an open reading frame encoding 417 amino acids. The sequences marked with the rectangle show the specific amplification primer pair for *ScCAX4*. * represents the stop codon. (**b**) The schematic diagram of the ScCAX4 protein conserved domains and transmembrane domains

As calcium-binding proteins, CAXs play vital roles in regulating the concentration of Ca^2+^ in cell compartments [27]. In this study, through reverse transcription-polymerase chain reaction (RT-PCR), one *CAX* gene (GenBank Acc No. MW206380) that encoded a polypeptide of 417 amino acids, was isolated from ROC22, a *Saccharum* spp. hybrid (Fig. 8a). Because ScCAX had a high homology with SsCAX4a (99.21 %) and SsCAX4e (99.13 %), both of which belong to the CAX family Type 1B (Table S10), this *ScCAX* was renamed *ScCAX4*. Analysis of the primary protein structure indicated that ScCAX4 is an acid unstable basic hydrophobic protein (Table S11). The phylogenetic tree showed that *ScCAX4* belongs to the Type 1B group, among which all members have a broad substrate range, and modulation of this transporter may be an important component of future strategies to improve plant ion tolerance. Pfam and transmembrane analyses showed that ScCAX4 contained two “Na_Ca_ex” domains (Pfam ID: PF01699). The “Na_Ca_ex” domain in the C-terminal denoted five core consensus transmembrane hydrophobic regions, and the one in the N-terminal represented another five core consensus transmembrane hydrophobic regions (Fig. 8b).

### Expression of ***ScCAX4*** in different tissues and under ABA, ***Sporisorium scitamineum***, and cold stresses

**Fig. 9:**
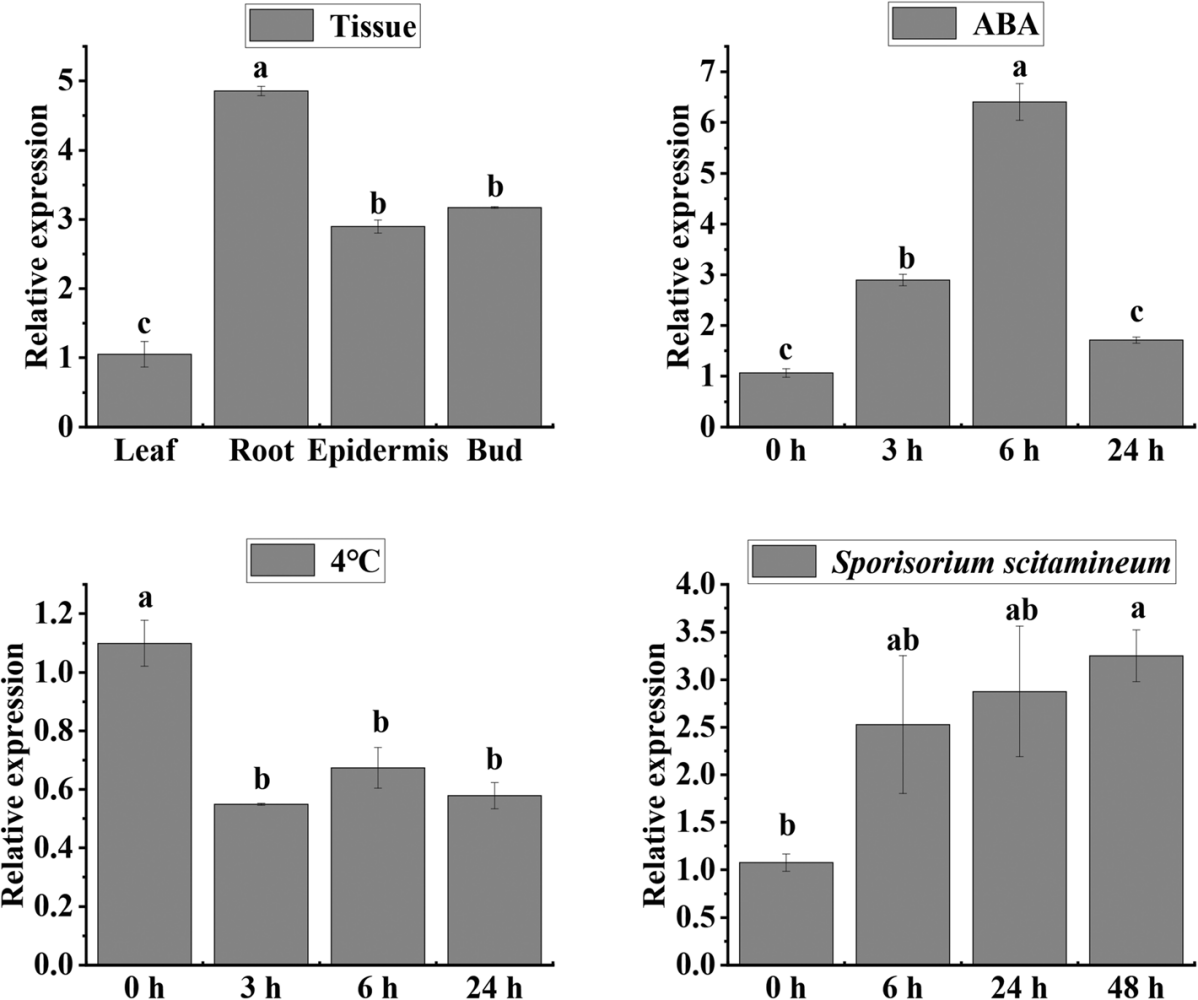
Expression patterns of the *ScCAX4* gene in sugarcane under ABA, *Sporisorium scitamineum*, and cold stresses. Error bars represent the standard error (SE) of three independent biological experimental repeats. The value on the Y-axis indicates the relative gene expression levels. The x-axis represents the time points when the samples were collected. Different lowercase letters indicate a significant difference, determined by one-way ANOVA, followed by Duncan’s new multiple range test (*p* < 0.05)

The expression of *ScCAX4* in four different sugarcane tissues, and under ABA, *S. scitamineum*, and cold (4 °C) stresses was detected by qRT-PCR (Fig. 9). *ScCAX4* was constitutively expressed in various tissues with different levels, and the expression in root was the highest. Under ABA treatment, the expression of *ScCAX4* was induced at 3 and 6 h. Under 4 °C stress, the transcript of *ScCAX4* was inhibited at all treatment time points. However, in response to *S. scitamineum* stress, the expression of *ScCAX4* was induced at all treatment time points.

### Subcellular localization of ScCAX4

**Fig. 10:**
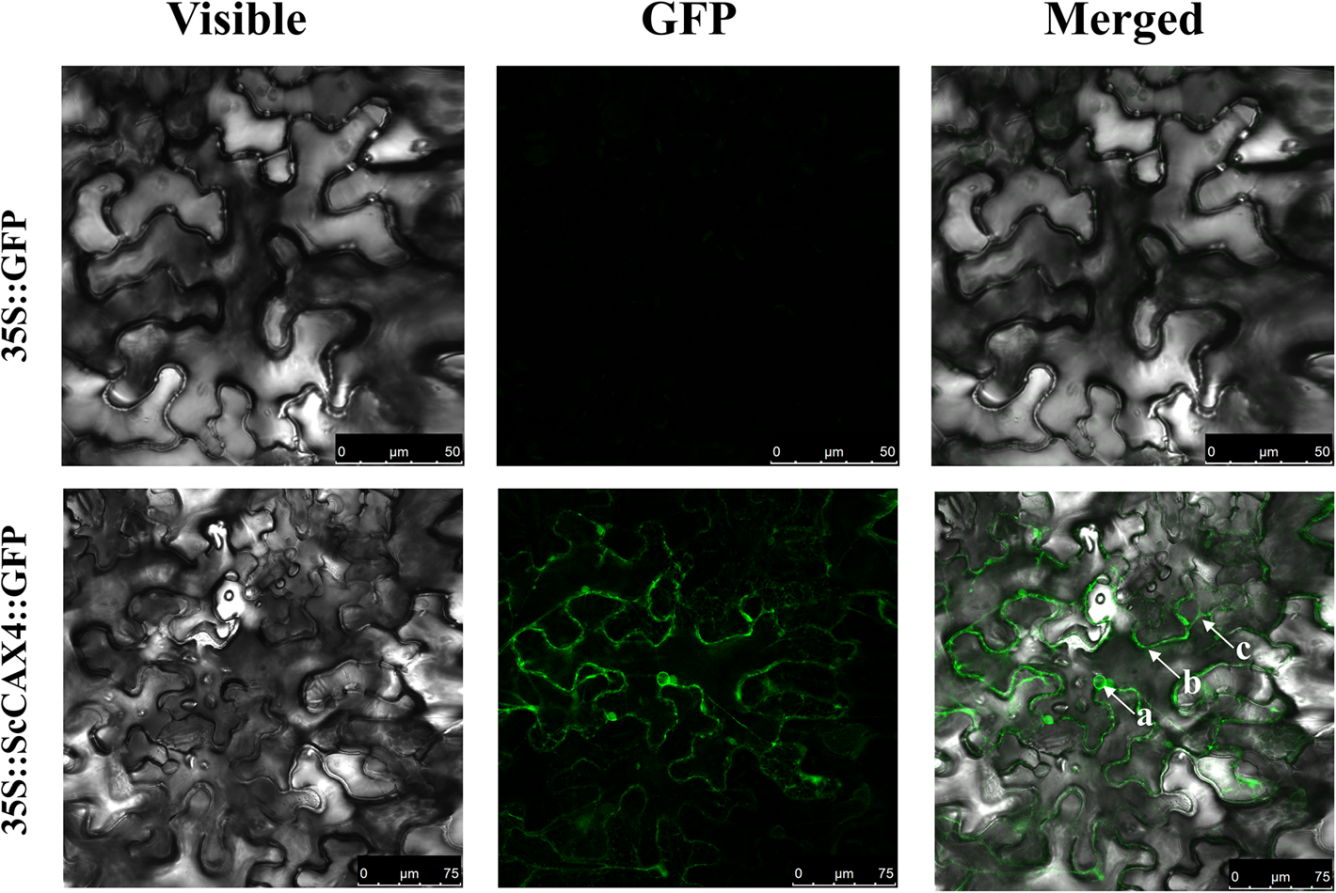
Subcellular localization analysis of ScCAX4 in *N. benthamiana* leaves. White arrow a, b, and c indicates the nucleus, plasma membrane, and cytoplasm, respectively. Epidermal cells were imaged using visible light, and green fluorescence, and merged images are shown. The scale bar is on the bottom right

The subcellular localization of the ScCAX4::GFP fusion protein was detected by laser scanning confocal microscopy after infiltration for two days. As indicated in Fig. 10, the ScCAX4::GFP fusion protein was localized in the cytoplasm, plasma membrane, and nucleus.

## Discussion

Members of the Ca^2+^/cation antiporter (CaCA) superfamily, as important “gatekeepers”, function in cellular ion homeostasis [13, 24]. At present, the CaCA superfamily genes have been identified and characterized in many plant species such as *A. thaliana* [15], *Zea mays* [8], and *Oryza sativa* [15]. However, virtually no systematic and comprehensive analyses of the *CaCA* superfamily genes in *S. spontaneum*, R570, or *S. bicolor* have been undertaken. In the present study, a multi-level analysis of *CaCA* genes from *S. spontaneum*, R570, and *S. bicolor* was performed to investigate their evolutionary relationships and functional divergence. The systematic characterization of the *CaCA* superfamily genes in the present study should provide a better foundation for further functional verification of this gene superfamily in the future.

### Evolution, conservation, and expansion of the CaCA superfamily

As phylogenetic analysis illustrated, these 53 CaCA superfamily genes from *S. spontaneum*, R570, and *S. bicolor* consisted of the CAX, CCX, EFCAX, and MHX families. Commonly, the CAX family genes are classified into two groups (Type 1A and Type 1B) [8]. The phylogenetic tree showed that 11 CAXs (eight SsCAXs and three SbCAXs) were clustered into Type 1A and eight CAXs (three SsCAXs, two ShCAXs, and three SbCAXs) were sorted into Type 1B. Type 1A CAX proteins, such as AtCAX1 and AtCAX3, are usually considered to be specific for Ca^2+^ [28, 29]. However, Type 1B CAX proteins, such as AtCAX2 and AtCAX5, can promote the transportation of various ions such as Ca^2+^, Cd^2+^, and Mn^2+^ [28, 30, 31]. In this study, we also divided 12 SsCCXs, one ShCCXs, and five SbCCXs into three categories according to the classification methods for AtCCXs [8]. In general, Group 1 and Group 2, which include CCXs from monocot and dicot plants, also contain CCXs from mosses. Group 3 contains solely angiosperm CCXs. Except for R570, within Group 3, there were usually two CCXs for each dicot species (*A. thaliana* and *Vitis vinifera*), three CCXs for each monocot species (*Brachypodium distachyon*, *Z. mays*, and *S. bicolor*), and nine CCXs for *S. spontaneum*, which clearly demonstrated recent gene duplication within Group 3.

Regarding their substrate specificity, it is tempting to speculate that members of Group 2 may share the function of AtCCX3 as an endomembrane H^+^-dependent K^+^ transporter [32], while plant members of Group 1 such as AtCCX5 may be more likely to have Ca^2+^ transport activity due to their closer relationship to NCKX6/NCLX [8]. Interestingly, the EFCAXs were divided into three groups (Group 1, Group 2, and Group 3). In addition, seven SsEFCAXs, one ShEFCAXs, and two SbEFCAXs were all clustered into Group 2, which only contained monocot EFCAXs, suggesting a diversification of EFCAX genes within land plants. With respect to the MHX family, there were some long branch lengths within the MHXs between angiosperms and mosses, indicating a degree of divergence between these MHXs. From the topology of the MHX phylogenetic tree, we can reasonably speculate that these MHXs in monocot and dicot plants evolved from the same ancestor.

### Potential functional roles of CaCA superfamily genes

In plants, miRNAs are involved in various crucial biological processes including plant development [33], biotic stress responses [34], abiotic stress responses [35], and signal transduction [36]. In our study, seven *SsCaCA* genes had four miRNA family target sites in *S. spontaneum*, while in *S. bicolor, ShCAX1* had two miRNA family target sites, and seven *SbCaCA* genes had 12 miRNA family target sites. Due to the more complete miRNA data for *S. bicolor*, the *SbCaCAs* matched more miRNAs. Interestingly, *SsCAX5a*, *SsCAX5b*, *SsCAX5c*, *ShCAX1*, *SbCAX4*, and *SbCCX4* all had the miR437 family target sites. Since members of miR437 can be found in *O. sativa*, *Z. may*, *S. bicolor*, and *Saccharum* spp., but not in *Arabidopsis* or *Populus*, miR437 is considered to be a monocot-specific miRNA [37, 38]. Several drought-related proteins, such as aquaporin, were found to be targeted by miR437 [39]. The results suggested that these *CaCA* genes may be involved in the drought stress response. Abdel-Ghany and Pilon et al. found that miR408 responds to copper deficiency in *Arabidopsis* [40]. *ShCAX1* and *SbCAX4* were both found to be targeted by miR408, which means that they may play the same role in the response to copper stress.

qRT-PCR analysis is commonly used to understand the function, especially the expression characteristics, of certain genes. The *CaCA* superfamily genes have been reported to play a role in various stresses in many plant species [9, 41, 42]. In the present study, the expression levels of *CaCA* genes under hormonal (ABA), biotic (*S. scitamineum*), and abiotic (cold) stresses were analyzed by qRT-PCR. As an important phytohormone, ABA plays a vital role in regulating plant growth, development, and stress responses [43]. Previous studies showed that Ca^2+^ channels were ABA-induced [44, 45]. In the present study, most of the candidate *CaCA* genes showed similar expression patterns and were upregulated at 3 or 6 h post ABA treatment. The results indicated that *CaCA* genes may play similar roles and function at the early stage of signal transduction after ABA treatment.

### Expression characteristics of the ***ScCAX4*** gene and the subcellular localization of the ScCAX4 protein

**Fig. 11:**
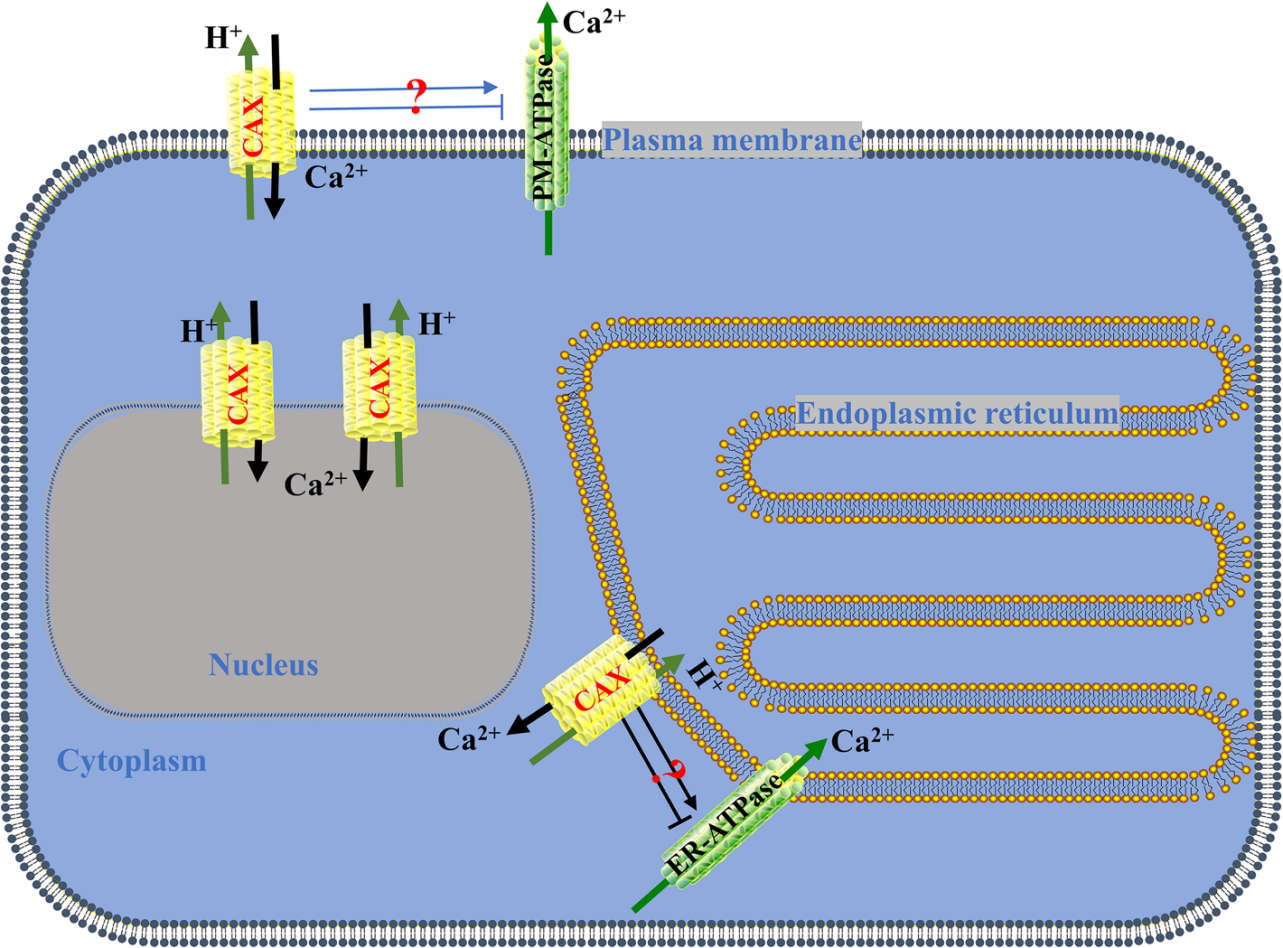
A hypothetical ion transport model of ScCAX4. PM-ATPase indicates plasma membrane-type calcium-transporting ATPase; ER-ATPase indicates endoplasmic reticulum-type calcium-transporting ATPase

*ScCAX4* is a member of the Type 1B group in the CAX family, suggesting that it may have a broad substrate range (including Ca^2+^, Cd^2+^, and Mn^2+^) [9]. *ScCAX4* was constitutively expressed in various tissues, with the highest expression in root. Under ABA treatment, the expression of *ScCAX4* was induced at 3 and 6 h. These results were similar to those observed for most of the *CaCA* genes in *Saccharum*, indicating their conserved function under ABA stress. Under *S. scitamineum* and 4 °C stress, *ScCAX4* exhibited two completely opposite expression trends and was inhibited and induced at all treatment time points under cold and *S. scitamineum* stresses, respectively. These results implied that *ScCAX4* may play contrasting roles in response to biotic and abiotic stresses. The subcellular localization showed that the ScCAX4::GFP fusion protein was localized in the cytoplasm, plasma membrane, and nucleus. Previous studies showed that most plant CAX proteins were determined to be localized to vacuolar membranes [30, 46, 47]. However, Ca^2+^/H^+^ exchange activity had been detected in plasma and plastid membranes [48, 49]. Furthermore, ScCAX4 contained 11 transmembrane domains. Based on the protein interactions among CaCA proteins, two proteins (SsCAX4a, and SsCAX4e), which had a high homology with ScCAX4, interacted with one plasma membrane-type calcium-transporting ATPase (Sb07g028160.1) and two endoplasmic reticulum-type calcium-transporting ATPases (Sb01g038990.1 and Sb09g001850.1). The results indicated that ScCAX4 may function as a calcium transporter (Fig. 11).

## Conclusions

In this study, 53 *CaCA* genes were identified from *S. spontaneum*, R570, and *S. bicolor*. These genes could be divided into four gene families: the CAX, CCX, EFCAX, and MHX families. The divergent biochemical characteristics of CaCA proteins were analyzed. Based on the phylogenetic tree, these CAX, CCX, EFCAX, and MHX family proteins had different classification statuses. The similar motif compositions of these proteins and the exon/intron structures of the *CaCA* genes within the families further supported the classification predicted by the phylogenetic tree. Synteny analysis and gene duplicated types of *CaCA* genes from *S. spontaneum*, R570, and *S. bicolor* provided valuable clues about their evolutionary characteristics. GO annotation and protein–protein interaction analysis revealed multiple functions for the CaCA proteins. The qRT-PCR analyses of the *SsCaCA* and *ShCaCA* genes confirmed that *CaCA* genes play a similar part under ABA treatment, but various roles in response to *S. scitamineum* and cold stresses. ScCAX4, a member of the Type 1B group in the CAX family, may function as a “transporter” and has a broad substrate range. Taken together, although the functions of these *CaCA* genes remain unknown and further research is still needed, our findings should lay a foundation for unraveling the biological roles and functions of *CaCA* genes in *Saccharum*.

## Methods

### Plant materials and treatments

The sugarcane cultivar ROC22 (*Saccharum* spp. hybrid) was provided by the Key Laboratory of Sugarcane Biology and Genetic Breeding, Ministry of Agriculture (Fuzhou, China).

For ABA treatment, sugarcane seedlings with 3–4 fully expanded leaves were treated with 100 µM ABA [50]. After treatment, the leaves were sampled at 0-, 3-, 6-, and 24-h time points. For *S. scitamineum* stress, the sugarcane cultivar ROC22 was inoculated with 0.5 µL of a 0.01 % (v/v) Tween-20 suspension containing 5 × 10^6^ smut spores·mL^− 1^; the control was inoculated with 0.01 % (v/v) Tween-20 in sterile distilled water. One mixed sample was collected for every three individual buds. Buds were randomly selected at 0 h, 6 h, 24 h, 48 h, and 120 h after inoculation [51]. For cold (4℃) treatments, the leaves were sampled at 0-, 6-, 24-, and 48-h time points [52–54]. Three biological replicates were prepared for each treatment. All the buds were frozen in liquid nitrogen and then stored at -80 °C until total RNA extraction.

### Total RNA extraction and first-strand cDNA synthesis

Total RNA was isolated using TRIzol reagent (Invitrogen, Shanghai, China) following the manufacturer’s instructions. The quantity and quality of RNA were assessed by a multifunction microplate reader Synergy H1 (Bio-Tek, Winooski, VT, USA) and 1.5 % agarose gel electrophoresis. The cDNA of RT-PCR was generated using 500 ng RNA with a HiScript III 1st Strand cDNA Synthesis Kit (+ gDNA wiper) (Vazyme, Nanjing, China) according to the manufacturer’s instructions. The cDNA of qRT-PCR was synthesized using 500 ng RNA with the HiScript Q RT SuperMix for qPCR (+ gDNA wiper) (Vazyme, Nanjing, China).

### Identification of ***CaCA*** superfamily genes

In this study, protein sequences and the genomic annotation of *S. spontaneum* were downloaded from the following link: http://www.life.illinois.edu/ming/downloads/Spontaneum_genome/ [17]. The monoploid reference R570 genome data (a single tiling path version) were obtained from the Sugarcane Genome Hub (http://sugarcane-genome.cirad.fr/) [25]. Phytozome (https://phytozome.jgi.doe.gov/) [55] was used to download *S. bicolor* data and the protein sequences of the other species.

To identify *CaCA* superfamily genes, the hidden Markov model (HMM) profile of Na_Ca_ex (PF01699) was downloaded from the Pfam protein family database (Pfam 32.0; http://pfam.sanger.ac.uk/) [56]. Hmmsearch (HMMer package version 3.1b1) was used to search Na_Ca_ex.hmm against the protein sequences from each plant genome [57]. If two or more transcripts were annotated for the same gene from alternative splicing, the first transcript isoform was selected for further study. The candidate CaCA proteins were then screened by hydropathy analysis using the TMHMM v2.0 program (http://www.cbs.dtu.dk/services/TMHMM/) to remove all non-TM proteins. Then, the remaining *CaCA* genes were further verified by the NCBI Conserved Domain Database (CDD: https://www.ncbi.nlm.nih.gov/cdd) [58] and Pfam (http://pfam.sanger.ac.uk/) .

### Multiple sequence alignment and phylogenetic analysis

Protein multiple sequence alignment (MSA) was analyzed by MUSCLE v3.7 with default parameters [59]. MEGA X was applied to infer the phylogenetic tree using NJ method with the following parameters: Poisson model, pairwise deletion, and 1000 bootstrap replications. IQ-TREE multicore version 1.6.12 was employed to construct ML tree with 1000 ultrafast bootstraps [60]. The resulting treefile was visualized with EvolView (https://www.evolgenius.info/evolview/#login) [61].

### Protein properties and sequence analyses

The basic properties (isoelectric point, molecular weight, grand average of hydropathicity, and instability index) of these CaCA proteins were predicted by ExPASy (http://web.expasy.org/protparam/). WoLF PSORT (https://wolfpsort.hgc.jp/) was used to predict the subcellular localizations of these CaCA proteins.

The protein motifs were analyzed by the MEME suite (http://meme-suite.org/tools/meme) with the following parameters: maximum number of motifs was 10, the optimum width of motifs was set between 10 and 50, and the distribution of motif occurrences was zero or one per sequence. The protein motifs and gene structures were illustrated by TBtools [62]. psRNATarget (http://plantgrn.noble.org/psRNATarget/) was applied to predict whether miRNA interacted with the *CaCA* genes [63].

### Synteny analysis and chromosome localization

The multiple Collinearity Scan toolkit (MCScanX) was used to detect the syntenic blocks and gene duplication events with the default parameters [64]. The syntenic blocks were used for constructing a synteny analysis map within and between genomes. Diagrams were generated using the Circos program (http://circos.ca/) and MCScanX [64]. Based on the comparative synteny map between the *S. spontaneum*, R570 or *S. bicolor* genomes, the Ks and Ka nucleotide substitutions between orthologous gene pairs were calculated by TBtools [62].

### Gene ontology annotation

The gene ontology terms for the CaCA proteins were identified using default parameters in the Blast2GO v5 tool [65]. Initially, the sequences were screened using BLASTP, followed by mapping, InterProScan analysis and annotation. Furthermore, the biological processes, cellular components and metabolic pathways were predicted using identified GO terms.

### Prediction of the protein–protein interaction network

The interaction network of CaCA proteins in *S. spontaneum*, R570, and *S. bicolor* was predicted using the STRING (version 11.0, http://string-db.org) website, which contains the known and predicted protein-protein interactions of different organisms [66]. In this study, all the CaCA protein sequences were submitted to the STRING website as queries, and *S. bicolor* was chosen as the reference organism for blasting because sugarcane has no protein–protein interaction database and is not included in STRING. After blasting, the matched homologs of *S. bicolor* with the highest scores (Bitscore) and more than 60 % identity were used to construct the network. The predicted interactions with scores > 0.4 (medium confidence level was set in STRING) are shown in the network.

### Expression patterns under ABA, ***Sporisorium scitamineum***, and cold stresses

qRT-PCR was used to detect the relative expression levels of nine *CaCA* genes (eight *SsCaCAs* and *ScCAX4*) under ABA, *S. scitamineum*, and cold stresses. The qRT-PCR primers of these *CaCA* genes were designed by Beacon Designer 8.14 software. The glyceraldehyde-3-phosphate dehydrogenase (*GAPDH* (GenBank Acc No. CA254672)) gene was chosen as the reference gene [67]. According to the ChamQ™ Universal SYBR^@^ qPCR Master Mix manual (Vazyme, Nanjing, China), the qRT-PCR reaction system was set as follows: SYBR Green Master Mix: 10 µL, 10 µM forward primers: 0.4 µL, 10 µM reverse primers: 0.4 µL, 10×diluted cDNA template: 1.0 µL, and sterile distilled water: 8.2 µL. The reaction procedure was as follows: 50 °C for 2 min, 95 °C for 10 min, 40 cycles of 95 °C for 15 s, and 60 °C for 1 min. The melting curves were analyzed after 40 cycles. The relative expression level of qRT-PCR data was calculated by standard curves (Supplementary Table S12) [68]. All primers used in qRT-PCR are listed in Supplementary Table S12. All sequences used in qRT-PCR are listed in Supplementary Table S13.

### Gene cloning and protein analysis

Based on our previous RNA sequencing data [69], the sequence of *ScCAX4* was obtained. The NCBI primer designing tool (http://www.ncbi.nlm.nih.gov/tools/primer-blast/) was used to design the cloning primers (Supplementary Table S12). RT-PCR with *LA* Taq (TaKaRa, Dalian, China) was used to amplify the *ScCAX4* from ROC22 [70]. The percentage identity between 11 SsCAXs, two ShCAXs, six SbCAXs and one ScCAX was calculated by Clustal Omega (https://www.ebi.ac.uk/Tools/msa/clustalo/). ExPASy (http://web.expasy.org/protparam/), the TMHMM v2.0 program (http://www.cbs.dtu.dk/services/TMHMM/), and Pfam protein family database (Pfam 32.0; http://pfam.sanger.ac.uk/) [56] were used to predict the primary protein sturctures, transmembrane domains and conserved domains, respectively.

### Subcellular localization analysis

The pFAST-R05-ScCAX4-GFP vector was constructed according to the gateway technology protocol. The induction medium (10 mM MES, 10 mM MgCl_2_, 200 µM acetosyringone, pH 5.0–5.4) was used to centrifuge and resuspend the *Agrobacterium tumefaciens* GV3101 cells, which contained pFAST-R05-ScCAX4-GFP, at an OD_600_ of 0.8. The *A. tumefaciens* GV3101 cells, which contained pFAST-R05-GFP, were used as a control. The induction medium was infiltrated into *Nicotiana benthamiana* leaves using a syringe without a needle. The subcellular localization results were visualized by laser scanning confocal microscopy (Leica TCS SP5, Wetzlar, Germany) after infiltration for two days.

## Supplementary Information

**Additional file 1:** **Figure S1** General characteristic features of the CaCA superfamily proteins in *S. spontaneum*, R570, and *S. bicolor*. Red triangles filled with white represent outliers, black diamonds filled with white represent the average, and different colored dots filled with black represent specific values. The detailed information in shown in Supplemental Table S1 and Table S2.**Figure S2** Phylogenetic evolutionary tree of the CaCA superfamily members. (a) An ML phylogenetic tree was constructed using the full-length sequence alignments of 47 CAX proteins. (b) An ML phylogenetic tree was constructed using the full-length sequence alignments of 43 CCX proteins. (c) An ML phylogenetic tree was constructed using the full-length sequence alignments of 28 EFCAX proteins. (d) An ML phylogenetic tree was constructed using the full-length sequence alignments of 12 MHX proteins. All the corresponding reference numbers are listed in Supplemental Table S1 and Table S3.**Figure S3** Phylogenetic relationship, architecture of conserved protein motifs, and structure of* CaCA* genes from *S. spontaneum*, R570, and *S. bicolor*. The phylogenetic tree was constructed based on the full-length sequences of CaCA proteins using MEGA X software. The motifs, numbers 1–10, are displayed in different colored boxes. The sequence information for each motif is provided in Supplemental Table S4. Green boxes indicate untranslated 5′- and 3′-regions, yellow boxes indicate exons, and black lines indicate introns. The length of the protein can be estimated using the scale at the bottom.**Figure S4** Sankey diagram showing the miRNA–mRNA network. Each rectangle represents a gene, and the connection of each gene is visualized based on the size of the rectangle.**Figure S5** Functional annotation of identified *CaCA* genes. The enrichment gene ontology analysis of *CaCAs *shows significantly enriched GO terms involved in biological processes (BP), molecular functions (MF), and cellular components (CC). **Figure S6** The melt curve plots of primers in this study.

**Additional file 2:** **Table S1**. The detailed information of *CaCA* genes included in this study.**Table S2**. General features of the CaCA superfamily proteins in *S. spontaneum*, R570 and *S. bicolor*.**Table S3**. GenBank accession numbers used in this study.**Table S4**. Analysis and distribution of conserved motifs in information CAX, CCX, EFCAX, and MHX family proteins from *S. spontaneum*, R570, and *S. bicolor*.**Table S5**. Collinearity relationships among *CaCA *genes.**Table S6**. The *CaCA *gene type in* S. spontaneum*, R570, and *S. bicolor*.**Table S7**. Orthologous relationships between *S. spontaneum*, R570, and *S. bicolor*.**Table S8**. The predicted miRNA target sites of *CaCA *genes in *S. spontaneum*, R570, and *S. bicolor.***Table S9**. Details of the GO distribution.**Table S10**. Percentage identity between 11 SsCAXs, two ShCAXs, six SbCAXs and one ScCAX was calculated using Clustal Omega.**Table S11**. Primary structure analysis of ScCAX4.**Table S12**. Primers used in this study.**Table S13**. All sequences used in qRT-PCR.

## Data Availability

The sequences of *ScCAX4* genes from sugarcane analysed during the current study are available in the NCBI repository with the Accession Numbers of MW206380. The data of *Saccharum spontaneum* genome can be downloaded from the following link: http://www.life.illinois.edu/ming/downloads/Spontaneum_genome/. The monoploid reference R570 genome can be downloaded from the following link: http://sugarcane-genome.cirad.fr/. The *Sorghum bicolor* genome can be downloaded from the following link: https://phytozome.jgi.doe.gov/. the protein sequences of the other species can be downloaded from the following links: https://phytozome.jgi.doe.gov/, https://www.ncbi.nlm.nih.gov/, and http://bioinformatics.psb.ugent.be/webtools/bogas/overview/Ectsi. The accession numbers are included in the supplemental data. All the other data supporting the conclusions of this article are within the paper.
