# Supplementary material for: The CaCA superfamily genes in Saccharum: comparative analysis and their functional implications in response to biotic and abiotic stress
Source: BMC Genomics. 2021 Jul 18;22:549. doi: 10.1186/s12864-021-07828-3 (PMC8286586; doi:10.1186/s12864-021-07828-3)
Supplement: Supplementary file 1 — Additional file 1: Figure S1 General characteristic features of the CaCA superfamily proteins in S. spontaneum, R570, and S. bicolor. Red triangles filled with white represent outliers, black diamonds filled with white represent the average, and different colored dots filled with black represent specific values. The detailed information in shown in Supplemental Table S1 and Table S2.Figure S2 Phylogenetic evolutionary tree of the CaCA superfamily members. (a) An ML phylogenetic tree was constructed using the full-length sequence alignments of 47 CAX proteins. (b) An ML phylogenetic tree was constructed using the full-length sequence alignments of 43 CCX proteins. (c) An ML phylogenetic tree was constructed using the full-length sequence alignments of 28 EFCAX proteins. (d) An ML phylogenetic tree was constructed using the full-length sequence alignments of 12 MHX proteins. All the corresponding reference numbers are listed in Supplemental Table S1 and Table S3.Figure S3 Phylogenetic relationship, architecture of conserved protein motifs, and structure of CaCA genes from S. spontaneum, R570, and S. bicolor. The phylogenetic tree was constructed based on the full-length sequences of CaCA proteins using MEGA X software. The motifs, numbers 1–10, are displayed in different colored boxes. The sequence information for each motif is provided in Supplemental Table S4. Green boxes indicate untranslated 5′- and 3′-regions, yellow boxes indicate exons, and black lines indicate introns. The length of the protein can be estimated using the scale at the bottom.Figure S4 Sankey diagram showing the miRNA–mRNA network. Each rectangle represents a gene, and the connection of each gene is visualized based on the size of the rectangle.Figure S5 Functional annotation of identified CaCA genes. The enrichment gene ontology analysis of CaCAs shows significantly enriched GO terms involved in biological processes (BP), molecular functions (MF), and cellular components (CC). Figure S6 The mel [file 12864_2021_7828_MOESM1_ESM.pdf]

The CaCA superfamily genes in *Saccharum*: comparative analysis and their functional implications in response to biotic and abiotic stress

Weihua Su[1,2], Chang Zhang[1,2] Dongjiao Wang[1,2], Yongjuan Ren[1,2], Tingting Sun[1,2], Jingfang Feng[1,2], Yachun Su[1,2], Liping Xu[1,2], Mutian Shi[3]* Youxiong Que[1,2]*

[1]Key Laboratory of Sugarcane Biology and Genetic Breeding, Ministry of Agriculture, Fujian Agriculture and Forestry University, Fuzhou 350002, Fujian, China

[2]Key Laboratory of Genetics, Breeding and Multiple Utilization of Crops, Ministry of Education, College of Crop Science, Fujian Agriculture and Forestry University, Fuzhou 350002, Fujian, China

[3]College of Horticulture, Fujian Agriculture and Forestry University, Fuzhou 350002, Fujian Province, China

***Correspondence should be addressed to shimutian@126.com, queyouxiong@126.com**

The full postal address of the submitting author Youxiong Que is as follows: Key Laboratory of Sugarcane Biology and Genetic Breeding, Ministry of Agriculture, Fujian Agriculture and Forestry University, Fuzhou 350002, China

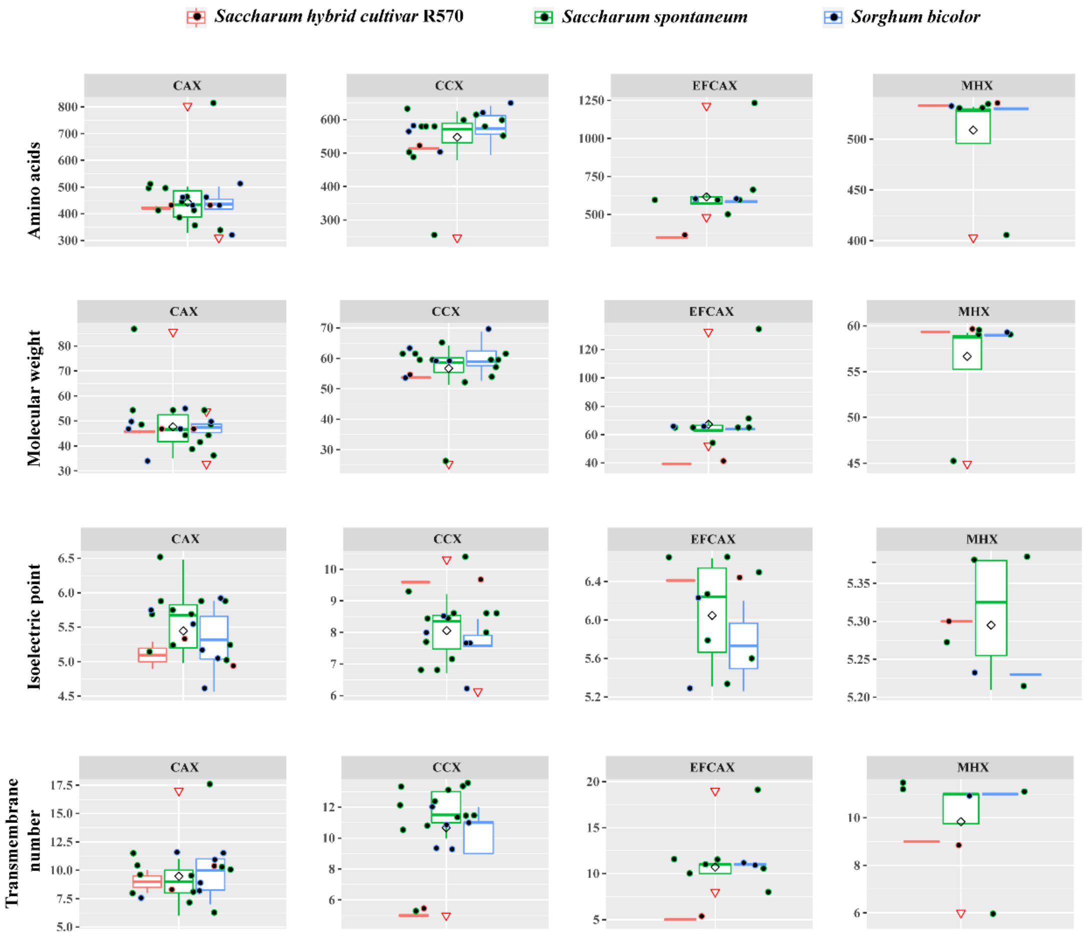

Fig. S1 General characteristic features of the CaCA superfamily proteins in *S. spontaneum*, R570, and *S. bicolor*. Red triangles filled with white represent outliers, black diamonds filled with white represent the average, and different colored dots filled with black represent specific values. The detailed information in shown in Supplemental Table S1 and Table S2.

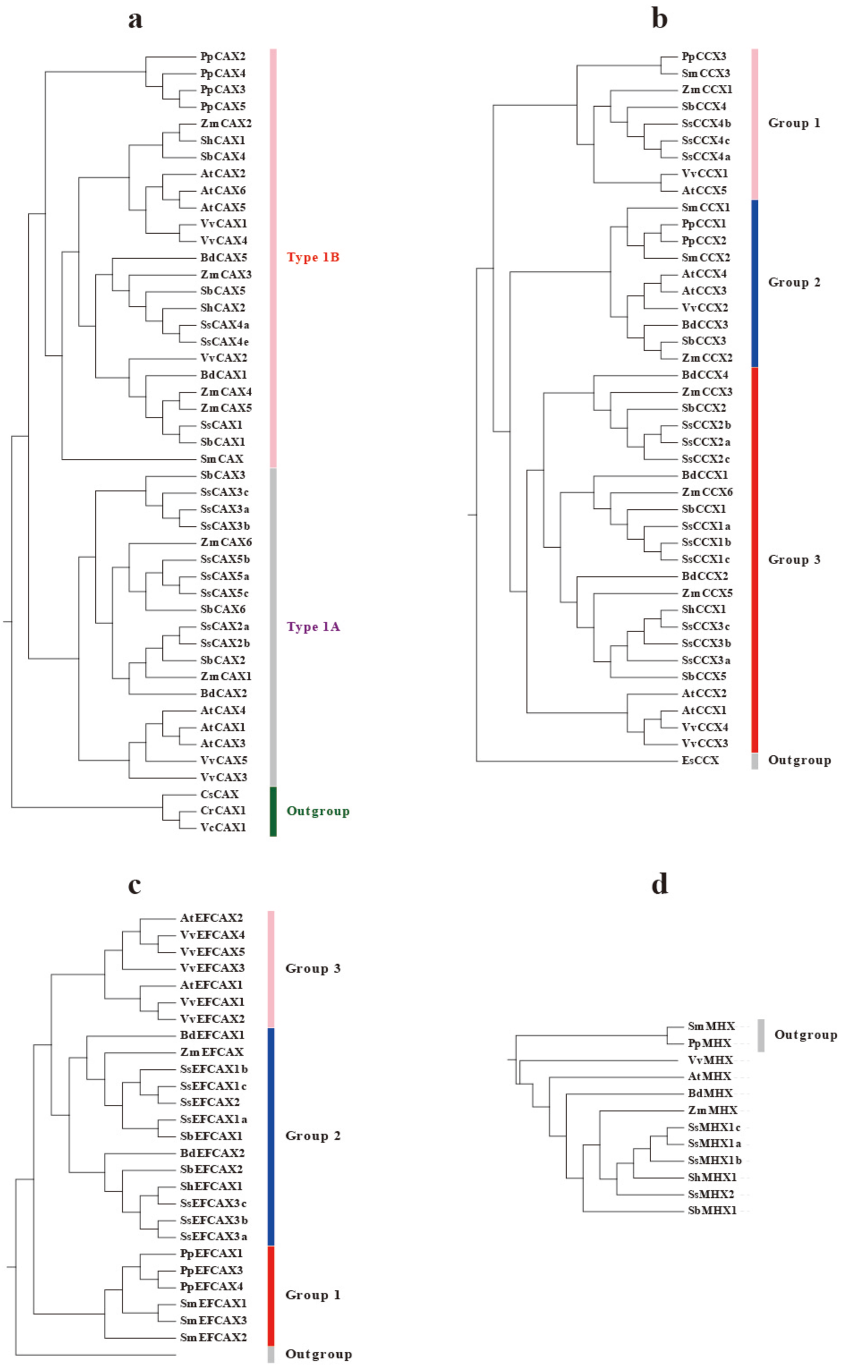

Fig. S2. Phylogenetic evolutionary tree of the CaCA superfamily members. (a) An ML phylogenetic tree was constructed using the full-length sequence alignments of 47 CAX proteins. (b) An ML phylogenetic tree was constructed using the full-length sequence alignments of 43 CCX proteins. (c) An ML phylogenetic tree was constructed using the full-length sequence alignments of 28 EFCAX proteins. (d) An ML phylogenetic tree was constructed using the full-length sequence alignments of 12 MHX proteins. All the corresponding reference numbers are listed in Supplemental Table S1 and Table S3.

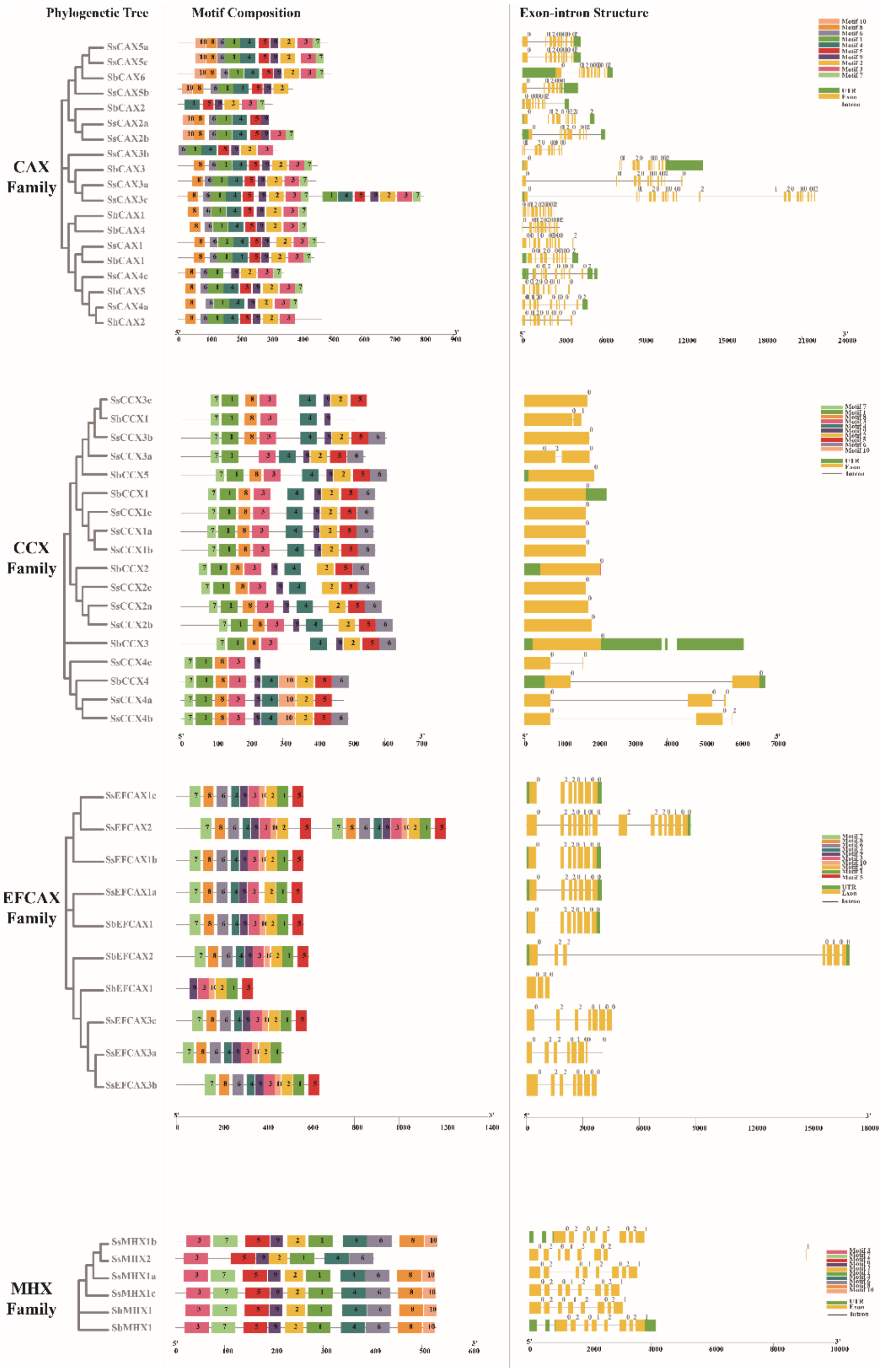

Fig. S3 Phylogenetic relationship, architecture of conserved protein motifs, and structure of *CaCA* genes from *S. spontaneum*, R570, and *S. bicolor*. The phylogenetic tree was constructed based on the full-length sequences of CaCA proteins using MEGA X software. The motifs, numbers 1–10, are displayed in different colored boxes. The sequence information for each motif is provided in Supplemental Table S4. Green boxes indicate untranslated 5′- and 3′-regions, yellow boxes indicate exons, and black lines indicate introns. The length of the protein can be estimated using the scale at the bottom.

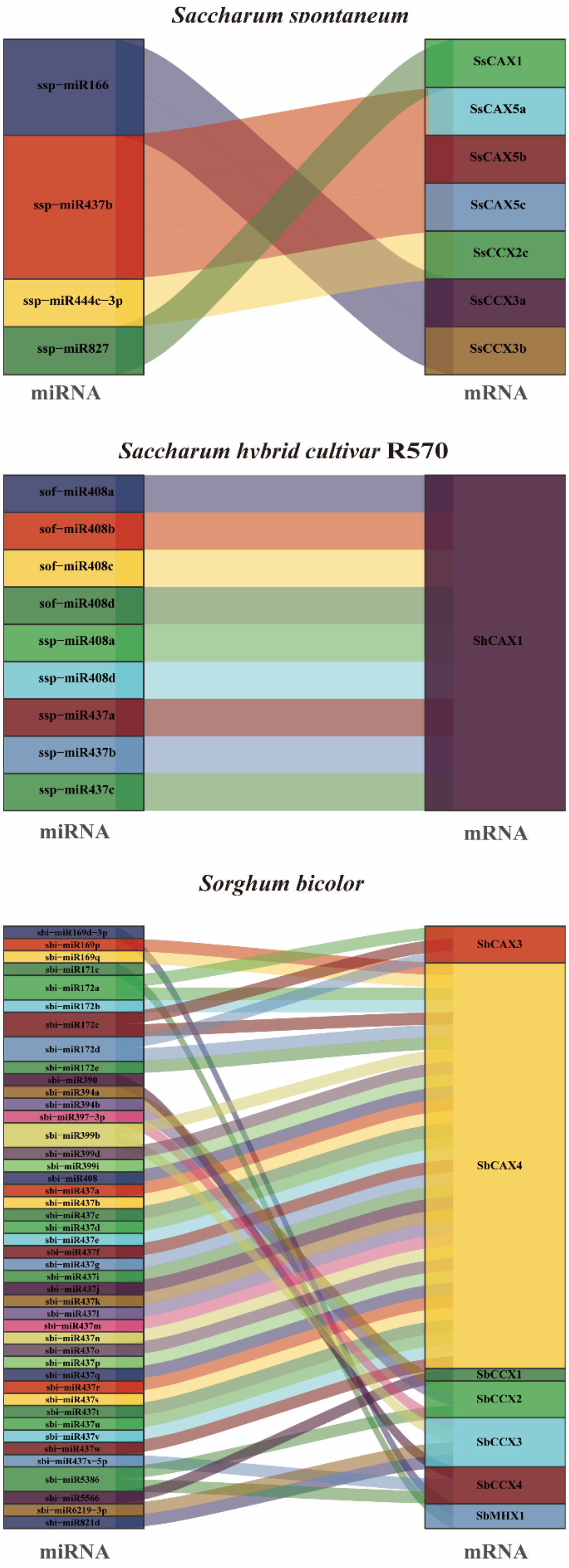

Fig. S4 Sankey diagram showing the miRNA–mRNA network. Each rectangle represents a gene, and the connection of each gene is visualized based on the size of the rectangle.

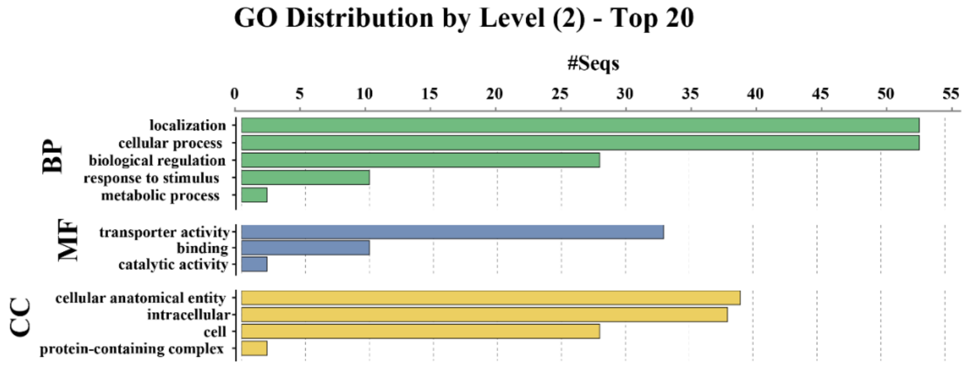

Fig. S5 Functional annotation of identified *CaCA* genes. The enrichment gene ontology analysis of *CaCAs* shows significantly enriched GO terms involved in biological processes (BP), molecular functions (MF), and cellular components (CC).

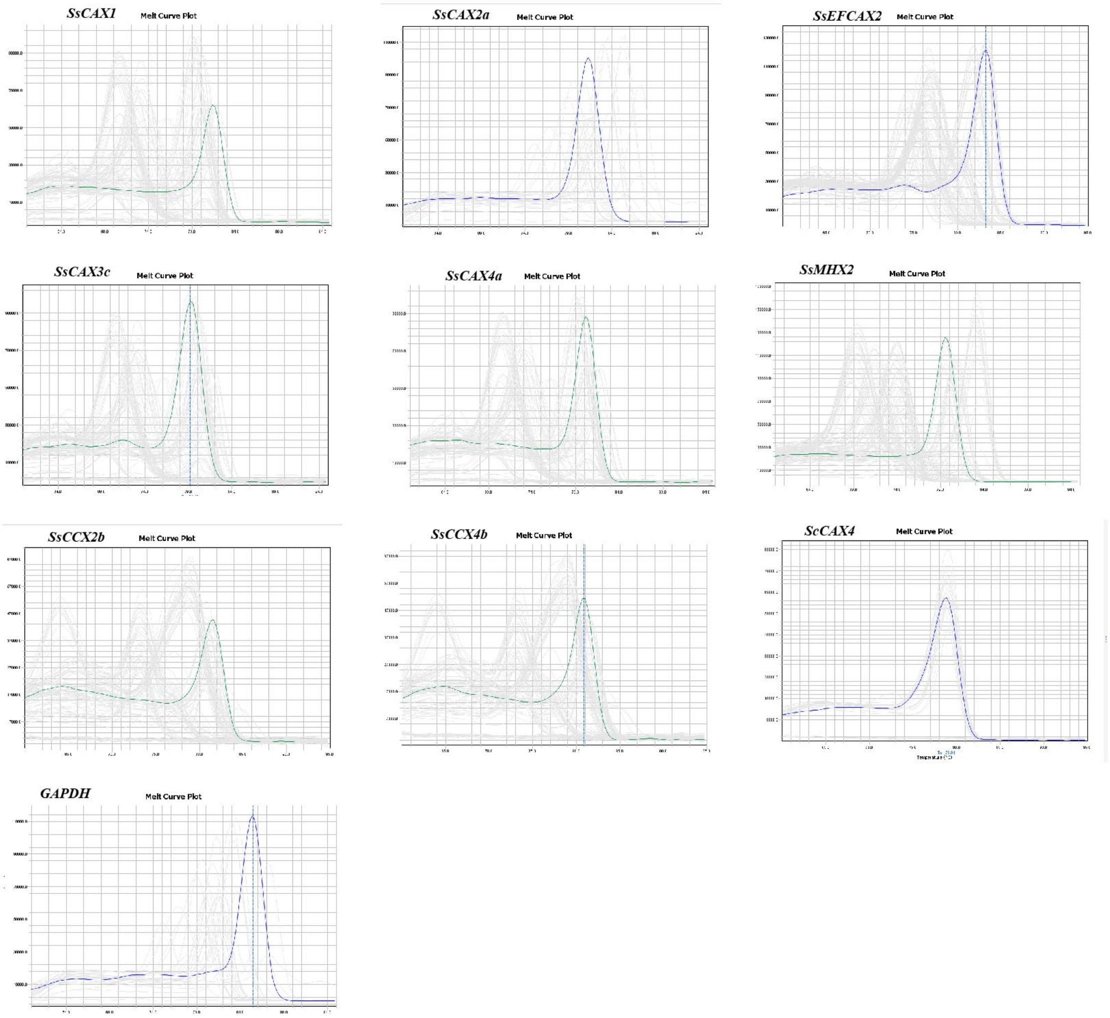

Fig. S6 The melt curve plots of primers in this study.
